# Effects of Neonicotinoid Pesticide Exposure on Human Health: A Systematic Review

**DOI:** 10.1289/EHP515

**Published:** 2016-07-06

**Authors:** Andria M. Cimino, Abee L. Boyles, Kristina A. Thayer, Melissa J. Perry

**Affiliations:** 1 Department of Environmental and Occupational Health, Milken Institute School of Public Health, George Washington University, Washington, DC, USA; 2 Office of Health Assessment and Translation, Division of the National Toxicology Program, National Institute of Environmental Health Sciences, National Institutes of Health, Department of Health and Human Services, Durham, North Carolina, USA

## Abstract

**Background:**

Numerous studies have identified detectable levels of neonicotinoids (neonics) in the environment, adverse effects of neonics in many species, including mammals, and pathways through which human exposure to neonics could occur, yet little is known about the human health effects of neonic exposure.

**Objective:**

In this systematic review, we sought to identify human population studies on the health effects of neonics.

**Methods:**

Studies published in English between 2005 and 2015 were searched using PubMed, Scopus, and Web of Science databases. No restrictions were placed on the type of health outcome assessed. Risk of bias was assessed using guidance developed by the National Toxicology Program’s Office of Health Assessment and Translation.

**Results:**

Eight studies investigating the human health effects of exposure to neonics were identified. Four examined acute exposure: Three neonic poisoning studies reported two fatalities (n = 1,280 cases) and an occupational exposure study of 19 forestry workers reported no adverse effects. Four general population studies reported associations between chronic neonic exposure and adverse developmental or neurological outcomes, including tetralogy of Fallot (AOR 2.4, 95% CI: 1.1, 5.4), anencephaly (AOR 2.9, 95% CI: 1.0, 8.2), autism spectrum disorder [AOR 1.3, 95% credible interval (CrI): 0.78, 2.2], and a symptom cluster including memory loss and finger tremor (OR 14, 95% CI: 3.5, 57). Reported odds ratios were based on exposed compared to unexposed groups.

**Conclusions:**

The studies conducted to date were limited in number with suggestive but methodologically weak findings related to chronic exposure. Given the wide-scale use of neonics, more studies are needed to fully understand their effects on human health.

**Citation:**

Cimino AM, Boyles AL, Thayer KA, Perry MJ. 2017. Effects of neonicotinoid pesticide exposure on human health: a systematic review. Environ Health Perspect 125:155–162; http://dx.doi.org/10.1289/EHP515

## Introduction

Neonicotinoids (neonics) are a class of chemicals used as insecticides for their neurotoxic action on the nicotinic acetylcholine receptor (nAChRs). Developed to replace organophosphate and carbamate insecticides, neonics are systemic in design, transfusing into all parts of treated plants, including pollen, nectar, and guttation fluids, and the foods grown by those plants (Jeschke et al. 2011; Chen et al. 2014). They are used for pest management across hundreds of crops in agriculture, horticulture, and forestry; in timber conservation and aquaculture; in vector control treatments for pets and livestock; and in urban and household pest control products (Simon-Delso et al. 2015). They are highly effective against difficult-to-control sucking, boring, and root-feeding insects (Goulson 2013).

The use of neonicotinoid insecticides in U.S. agricultural production has grown dramatically in the past decade (Douglas and Tooker 2015; Hladik et al. 2014; Jeschke et al. 2011; Simon-Delso et al. 2015). In conjunction with an industry shift toward prophylactic application of pesticides, the sale of seeds pretreated with neonics tripled from 2004 to 2014 (Haire 2014; Hladik et al. 2014). Currently more than 90% of all corn and 44–50% of soybeans are grown from seeds coated with neonics, and they are used extensively on other cereal and oil crops and fruit and vegetables as well (Aginfomatics 2014; Chen et al. 2014; Douglas and Tooker 2015; Hladik et al. 2014; Krupke et al. 2012; Simon-Delso et al. 2015). Neonics are also applied later in the growing cycle via drip and broadcast and foliar spraying (van der Sluijs et al. 2015; Chen et al. 2014). In the United States, it is estimated that more than 4 million pounds of neonics are applied to between 140 and 200 million acres of cropland annually (Douglas and Tooker 2015; Center for Food Safety 2014). The value of neonic treated seeds alone is worth approximately $1.4 billion to the U.S. economy. Based on current trends, neonic use is likely to increase due to expanded application of seed treatments for crops in which they are not yet predominant (e.g., soybeans and wheat) and a change in the “standard” seed treatment from the lowest (0.25 mg/seed) to the highest allowable rate (1.25 mg/seed) (Douglas and Tooker 2015).

Neonics are persistent in the environment: They have been found in soil, dust, wetlands, ground water, nontarget plants and vertebrate prey, and foods common to the American diet, including wild and aqua cultured marine species (Anderson et al. 2015; Bonmatin et al. 2015; Chagnon et al. 2015; Chen et al. 2014; Cycoń and Piotrowska-Seget 2015; FDA 2014; Hladik et al. 2014; Huseth and Groves 2014; Koshlukova 2006; Krupke et al. 2012; Main et al. 2014; Simon-Delso et al. 2015; USDA 2014).

The U.S. Department of Agriculture’s (USDA) 2014 pesticide monitoring report found neonics in 12 of 19 different fruits and vegetables sampled, with 11 of these containing multiple neonics, an increase compared to the previous USDA PDP report, which reported neonics were detected in 11 of 17 fruits and vegetables, with only two containing multiple neonics (USDA 2014, 2016).

The USDA reported levels in one food (summer squash) exceeded the maximum residue limit (MRL) for thiamethoxam (THX) (USDA 2014). A study using more sensitive analytical techniques than those used by the USDA prior to 2013 also reported finding multiple neonics in several fruits and vegetables (seven apple varieties, oranges, cantaloupe, and spinach) and in five organic honey samples (Chen et al. 2014). In its 2012 Total Diet Study, the FDA reported neonics were among the most frequently found pesticide residues in infant and toddler foods (occurrence ranging from 6% to 31%) (FDA 2015). Unlike most other pesticides, neonics cannot be washed off of food prior to consumption (Chen et al. 2014).

When the U.S. Environmental Protection Agency (EPA) first approved neonics for commercial use, they were considered less toxic to wildlife and humans because of a higher chemical affinity for insect nAChRs and an inability to cross the mammalian blood–brain barrier (Tomizawa and Casida 2003). Although the studies required for pesticide registration showed neonics to be less toxic to mammals than to insects, toxic effects such as an increase in cancerous liver tumors in mice were noted (U.S. EPA 2000; Gibbons et al. 2015), supporting the U.S. EPA’s establishment of MRLs for the leading neonics used in American agriculture: imidacloprid (IMI), clothianidin (CLO), THX, and acetamiprid (ACE).

Neonics have since been linked to adverse effects in vertebrate as well as invertebrate species (Gibbons et al. 2015; Goulson et al. 2015; Krupke et al. 2012; Mason et al. 2013; Morrissey et al. 2015; Pisa et al. 2015; Rundlöf et al. 2015; Sánchez-Bayo 2014; Whitehorn et al. 2012; van der Sluijs et al. 2015). More recent *in vitro* and *in vivo* studies as well as ecological field studies indicate neonics can have adverse effects on mammals, including at sublethal doses (Calderón-Segura et al. 2012; Gibbons et al. 2015; Gu et al. 2013; Kimura-Kuroda et al. 2012; Mason et al. 2013). Certain neonic metabolites have been found to be as or more toxic than the parent compound (Chen et al. 2014; Goulson et al. 2015; Simon-Delso et al. 2015; Tomizawa 2004). One of IMI’s breakdown products, desnitro-imidacloprid, for instance, has a high affinity for mammalian nAChRs, is known to be highly toxic to mice (Chao and Casida 1997), and can be formed either in a mammal’s body during metabolism or in the environment (Koshlukova 2006).

Neonics have been found to affect mammalian nAChRs in a way that is similar to the effects of nicotine (Kimura-Kuroda et al. 2012). These receptors are of critical importance to human brain function, especially during development (Kimura-Kuroda et al. 2012) and for memory, cognition, and behavior (Chen et al. 2014). A distinct aspect of neonic toxicity is the ability to bind to the most prominent subtype of nAChRs in mammals, the α4β2, which is found in the highest density in the thalamus (Chen et al. 2014; Li et al. 2011). Alteration of the density of this neuroreceptor subtype has been found to play a role in several central nervous system disorders, including Alzheimer’s disease, Parkinson’s disease, schizophrenia, and depression. In the developing brain, this subtype is involved in neural proliferation, apoptosis, migration, differentiation, synapse formation, and neural circuit formation (Chen et al. 2014). Other studies have found adverse reproductive as well as developmental effects in mammals including reduced sperm production and function, reduced pregnancy rates, higher rates of embryo death, stillbirth, and premature birth, and reduced weight of offspring (Abou-Donia et al. 2008; Gibbons et al. 2015; Gu et al. 2013).

The goal of this systematic review was to identify relevant human population studies on the health effects of neonicotinoids. Specific aims included evaluating the risk of bias (internal validity) of relevant studies, determining the extent to which findings could be synthesized across studies to reach level-of-evidence conclusions (NTP 2015) for any associations reported between neonicotinoids and human health, and addressing research implications based on that evidence.

## Methods

### Research Question

A PICO (population, intervention, comparator, outcome)/PECO (population, exposure, comparator, outcome) statement was developed to address and understand potential effects of neonics on humans (NTP 2015) (Table 1). The PECO statement was used to define the research question and develop the search terms and inclusion and exclusion criteria for the systematic review.

**Table 1:** PECO (population, exposure, comparator, outcome) statement.

PECO	Evidence
Population	Humans of all ages (including prenatal)
Exposure	Neonic pesticides at any concentration
Comparator	A comparison group exposed to lower levels (or no exposure or exposure below detection levels) compared to more highly exposed participant.
Outcome	Any health effect

### Search Design

The peer-reviewed literature published in English between January 2005 and November 2015 was searched for relevant studies. This period was chosen as it overlaps with the sharp increase in prophylactic use of neonics in U.S. agriculture, particularly neonic-coated seeds and soil injections.

Articles were primarily identified from database searches in PubMed, Scopus, and Web of Science. In addition, the reference lists of relevant records were searched to capture articles that may have been missed in the database searches. The following search terms were used: “neonicotinoids AND human health”; *“*imidacloprid OR clothianidin OR thiamethoxam OR acetamiprid AND human health”; “neonicotinoids AND occupational exposure/adverse effects”; “neonicotinoids AND environmental exposure/adverse effects”; “neonicotinoids AND maternal exposure”; “neonicotinoids AND prenatal exposure”; “neonicotinoids AND migrants and transients”; “neonicotinoids AND neurological development”; “neonicotinoids AND fetal development”; “neonicotinoids AND teratogenicity”; “neonicotinoids AND bioaccumulation”; “neonicotinoids AND biomagnification”; “neonicotinoid metabolites AND human health”. All terms were searched using both controlled vocabulary [Medical Subject Headings (MeSH) in PubMed] and free text words in titles and abstracts.

To be eligible for inclusion, studies needed to comply with the criteria specified by the PECO statement (Table 1). Studies that did not meet the PECO criteria were excluded. In addition, the following exclusion criteria were applied:

Studies did not contain original data, such as reviews, editorials, or commentaries.
Studies were not peer-reviewed (e.g., conference abstracts, technical reports, theses and dissertations, working papers from research groups or committees, and white papers).
Animal toxicological studies.
Molecular studies and assay tests of human tissues, cells, and genes.
Individual medical case studies.
Studies did not assess neonic exposure separately from other pesticide classes.

### Data Collection

We collected the following data for each study: authors, journal, year of publication, country, study design, study population, exposure assessment, health outcome diagnosis, measures of association, adjustment factors, and other critical comments.

### Assessing Study Quality

Study quality was assessed using a protocol developed by the Office of Health Assessment and Translation (OHAT) (NTP 2015). Risk of bias (RoB) in methodology was assessed by answering up to nine questions, based on type of study. The RoB questions covered biases in subject selection, quality of exposure assessment, attrition or exclusion of subjects, detection of outcomes, selective reporting of outcomes, and statistical methodology. Questions were rated as “definitely low RoB,” “probably low RoB,” “probably high RoB,” or “definitely high RoB.” Table 2 depicts the questions and RoB ratings for the studies in this review. Several studies were retained in this review despite being assessed as having “probably to definitely high risk of bias,” as well as other factors reducing the level of confidence in their findings, in order to explore the knowledge base to date for human health effects to chronic (versus acute) neonic exposure. Because of the small number of heterogeneous studies and disparate outcomes assessed we did not conduct a meta-analysis or attempt to rate confidence across the body of studies.

**Table 2:** Risk of bias analysis: neonics and human health.

Reference	Comparison groups appropriate	Confounding/modifying (design/analysis)	Identical experimental conditions	Blinding subjects and researchers	Outcome data complete	Exposure characterization confidence	Outcome assessment confidence	All measured outcomes reported	Other validity/statistical issues
Source: acute studies
Elfman et al. 2009	++	–	+	++	++	–	–	++	–
Forrester 2014	NA	––	NA	NA	NA	––	––	+	–
Mohamed et al. 2009	NA	+	NA	NA	–	–	+	++	–
Phua et al. 2009	NA	++	NA	NA	NA	–	+	++	–
Source: chronic studies
Carmichael et al. 2014	++	–	NA	NA	–	–	–	+	–
Keil et al. 2014	+	–	NA	NA	–	+	–	–	–
Marfo et al. 2015	++	–	NA	NA	–	+	+	+	–
Yang et al. 2014	++	–	NA	NA	–	–	–	+	–
Note: ++, definitely low risk of bias; +, probably low risk of bias; ––, definitely high risk of bias; –, probably high risk of bias; NA, not applicable. Overall rating: Tier 3, probably high risk of bias, low to moderate confidence. Under OHAT, all chronic studies would be dropped as too weak for inclusion, as would the most recent (2014) acute study. All were retained to enable this review.									

## Results

**Figure 1:**
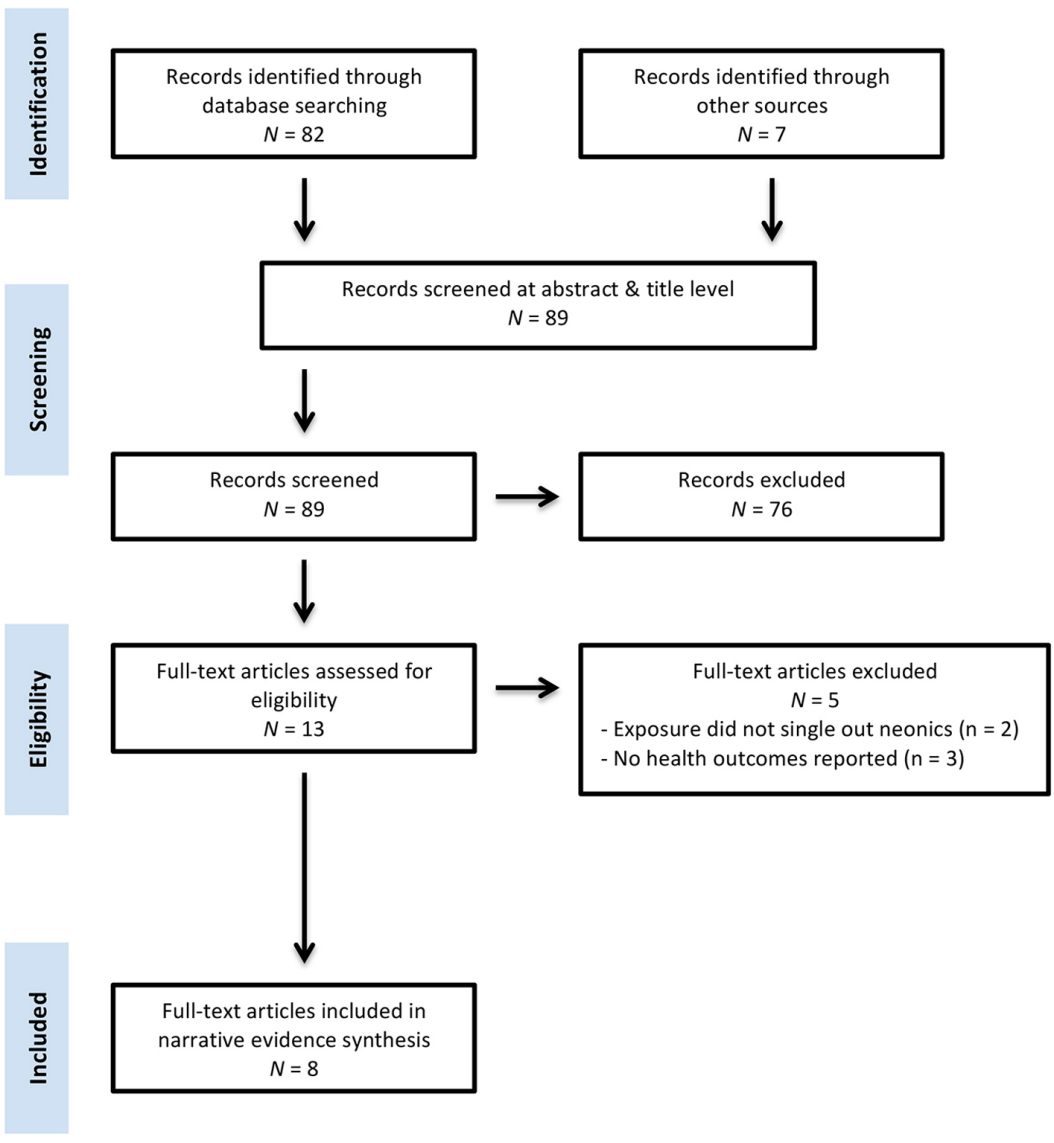
Neonics and human health study selection flow diagram.

In this systematic review, 89 unique references were identified (see “Neonics and Human Health: All References” in the Supplemental Material for a complete list). Of these, 76 were excluded on the basis of title and abstract. Of the 13 remaining, after a critical review of the full text, 5 more studies were excluded because they did not report human health effects or outcomes (Cao 2015; Craig 2005; Hou et al. 2013) or because they did not assess the effects of neonic exposure as a separate class from other pesticides (Khan et al. 2008, 2010). Figure 1 provides the study selection flow diagram for this review.

Table 3 summarizes the eight studies investigating neonics and human health included in this review, organized by type of exposure, either acute or chronic (i.e., nonacute). Three examined the acute health effects of neonic poisonings, including the clinical outcomes of self-poisoning (Forrester 2014; Mohamed et al. 2009; Phua et al. 2009), and one study analyzed the health effects of acute occupational neonic exposure (Elfman et al. 2009). The other four studies analyzed the health effects of chronic (i.e., nonacute) environmental exposure to neonicotinoids (Carmichael et al. 2014; Keil et al. 2014; Marfo et al. 2015; Yang et al. 2014).

**Table 3:** Summary of studies investigating neonic exposure and adverse human health effects (January 2005–December 2015).

Studies	Study population	Country of study	Results
Acute exposure
Elfman et al. 2009	19 conifer seedling planters: 17 men, 2 women	Sweden	No clear acute adverse effects reported after 1 week of exposure to IMI-treated seedlings.
Forrester 2014	1,142 exposure cases reported to a TX poison control network from 2000 to 2012	USA	Of the 1,142, 77% were identified as IMI alone or in combination with other neonics. Thirty-two neonic exposures (2.9%) resulted in “serious medical outcomes” including ocular irritation/pain, dermal irritation/pain, nausea, vomiting, oral irritation, red eye, erythema, rash, numbness, and dizziness. Chest pain (2 exposures; 0.2%), hypertension (0.2%), and tachycardia (0.2%) were the most frequently reported serious cardiovascular effects. No deaths reported.
Mohamed et al. 2009	68 hospital patients: 61 ingestion, 7 dermal exposures	Sri Lanka	Of the 56 patients with acute IMI poisoning (versus mixtures), only 2 developed severe symptoms. The majority had mild symptoms including nausea, vomiting, headache, dizziness, abdominal pain, and diarrhea. IMI exposure confirmed in 28 cases, with a median plasma concentration of 10.58 ng/L (IQR: 3.84–15.58 ng/L; range: 0.02–51.25 ng/L) on admission. Concentrations for 7 patients remained elevated for 10–15 hr post-ingestion, suggesting absorption and/or elimination may be saturable or prolonged at high doses. No deaths reported.
Phua et al. 2009	70 exposure cases reported to the Taiwan National Poison Center	China	Of the 57 cases of ingested neonics, the majority were of IMI (*n* = 53), followed by ACE (*n* = 2) and CLO (*n* = 2). The 10 most severe cases were from IMI alone. Two deaths reported (mortality rate 2.9%).
Chronic exposure
Carmichael et al. 2014	101 heart defect cases recruited from mothers who participated in a pop-based case control study in San Joaquin valley; 9 exposed/92 not exposed	USA	Significant association between residential proximity to agricultural use of IMI and tetralogy of Fallot (AOR 2.4, 95% CI: 1.1, 5.4).
Keil et al. 2014	407 children with ASD recruited from Childhood Autism Risks from Genetics and Environment (CHARGE) Study/206 controls	USA	Weak association between prenatal exposure to IMI and ASD (AOR 1.3, 95% CrI: 0.78, 2.2); OR increased to 2.0 (95% CrI: 1.0, 3.9) when limiting study population to those who self-identified as “frequent users” of flea and tick medicines containing IMI.
Marfo et al. 2015	35 symptomatic cases in Gunma prefecture/50 controls	Japan	Significant association between urinary DMAP and increased prevalence of memory loss, finger tremor, and other symptoms of unknown origin (OR 14, 95% CI: 3.5, 57).
Yang et al. 2014	73 anencephaly cases in San Joaquin valley; 6 exposed/67 not exposed	USA	Suggestive association between residential proximity to agricultural use of IMI and anencephaly (AOR 2.9, 95% CI: 1.0, 8.2).
Note: ACE, acetamiprid; AOR, adjusted odds ratio; ASD, autism spectrum disorder; CI, 95% confidence interval; CLO, clothianidin; CrI, credible interval; IMI, imidiacloprid.			

### Acute Exposure Studies Reporting No Adverse Health Effects

One of the four acute exposure studies reported no adverse health effects associated with the neonic of interest (IMI) and no clear correlations between reported symptoms and exposure to IMI (Elfman et al. 2009). The investigation, a double-blind crossover study in which cases served as their own controls, followed 19 planters of conifer seedlings treated with either IMI or another insecticide or left untreated. Elfman et al. (2009) relied on both questionnaire and biomonitoring data (nasal mucous and urine).

### Acute Exposure Studies Reporting Adverse Health Effects

Three of the four acute exposure studies—two retrospective analyses of poison control center data (Forrester 2014; Phua et al. 2009) and one prospective observational cohort following hospital patients with confirmed IMI poisoning (Mohamed et al. 2009)—looked at a total of 1,280 neonic exposures. Of these, 698 were oral ingestions, with 582 exposed via other pathways (dermal, ocular, inhalation, injection, otic exposure, or unspecified).

Because there is no antidote to neonic poisoning in mammals (Forrester 2014), any ingestion was considered acute by this review.

The three poisoning studies all reported IMI was the most common neonic used in self-poisonings (*n* = 884 IMI; *n* = 99 IMI in combination with other chemicals). Cases of self-poisoning with ACE (*n* = 8), THX (*n* = 6), and CLO (*n* = 5) were few in comparison (Phua et al. 2009; Forrester 2014).

Of the four acute exposure studies, only one reported fatalities (*n* = 2) following acute exposure to IMI (Phua et al. 2009). This result was based on analysis of 70 neonic poisonings reported to the Taiwan National Poison Center from 1987 to 2007, of which 46 were cases of neonic ingestion alone. The other 24 cases were coexposed to a different class of pesticide and/or ethanol. Ten of the 46 neonic-only cases developed severe symptoms, defined as requiring intubation and intensive care, versus 36 who were asymptomatic or had mild to moderate symptoms and required only supportive care. Two of the severely ill group died of respiratory failure. All 10 who developed severe/fatal poisoning ingested only IMI. The difference between the amounts ingested by the severe/fatal poisoning group versus the non-severe group was not significant (*p* = 0.938), suggesting either exposure misclassification or factors other than IMI exposure contributed to severity/fatality.

Forrester (2014) reported a serious outcome rate of 2.9% (32 cases out of 1,095 total neonic exposures excluding those with a medical outcome of unrelated effects). Forrester (2014) defined serious outcome as “moderate effect, major effect, death, and unable to follow but judged as potentially toxic exposure.” Moderate effect in Forrester (2014) included symptoms the other poisoning studies defined as “mild,” such as dermal and ocular irritation. All three poisoning studies reported cardiovascular effects were a rare but possible serious clinical outcome of acute neonic exposure. Aspiration pneumonia and respiratory failure were found to be significant complications by Phua et al. (2009). None of the studies reported seizures or rhabdomyolysis as outcomes of acute neonic exposure. Two poisoning studies reported acute neonic ingestion produced symptoms similar to acute organophosphate or carbamate poisoning (Phua et al. 2009; Mohamed et al. 2009). Both studies warned the antidotes for these pesticide classes (oximes and atropine) should not be used as treatments for neonic poisonings as they may worsen outcomes.

Only one study addressed the toxicokinetics of IMI poisoning. Mohamed et al. (2009) reported that concentrations of IMI remained elevated for up to 10–15 hr post-ingestion, suggesting humans have a saturable (zero order) absorption and elimination ability for high doses of IMI. The authors noted their toxicokinetic findings would have been better defined had a quantification of metabolic production in humans been available because variation in cytochrome P450 isoenzymes involved in oxidative IMI metabolism may contribute to variable toxicity. Forrester (2014) suggested neonics might differ in their levels of toxicity to humans, observing the serious outcome rate for IMI poisoning was higher than for dinotefuran or nitenpyram.

### Chronic Exposure Studies Reporting No Adverse Health Effects

None. All four case–control studies reported an association between chronic (i.e., nonacute) neonic exposure and an adverse human health effect.

### Chronic Exposure Studies Reporting Adverse Health Effects

Four studies identified in this review reported an association between chronic environmental exposure to IMI, THX, or *N*-desmethyl-acetamiprid (DMAP), a metabolite of ACE, and an adverse human health effect (Carmichael et al. 2014; Keil et al. 2014; Marfo et al. 2015; Yang et al. 2014). Three of the studies focused on developmental health outcomes, including congenital heart defects (CHDs) (Carmichael et al. 2014), neural tube defects (NTDs) (Yang et al. 2014), and autism spectrum disorder (ASD) (Keil et al. 2014). Two of the developmental outcome studies focused on maternal residence proximity to agricultural use of pesticides during periconception as the exposure pathway (Carmichael et al. 2014; Yang et al. 2014); the third examined maternal use of flea and tick medication containing IMI from 3 months before conception through 3 years of age (Keil et al. 2014). In Marfo et al. (2015) exposure was based on urine samples collected from a patient population presenting with a specific cluster of symptoms associated with neonic exposure, including neurological outcomes (memory loss, finger tremor) and at least five of six other health measures; diet questionnaire data; and residential proximity to agricultural use of neonics.

Total sample sizes included 407 cases of ASD (262 controls) (Keil et al. 2014); 569 heart defect cases (785 controls) (Carmichael et al. 2014); 650 cases of NTDs (785 controls) (Yang et al. 2014); and 35 symptomatic cases (50 controls) (Marfo et al. 2015). Sample sizes for the CHD and NTD phenotypes associated with neonic exposure were smaller: tetralogy of Fallot (*n* = 101 cases) and anencephaly (*n* = 72) (Carmichael et al. 2014; Yang et al. 2014). The sample size of those with “typical symptoms” associated with DMAP exposure (versus those with “atypical symptoms” *n* = 16) was also small (*n* = 19) (Marfo et al. 2015). Three of the four chronic exposure studies reported findings related only to IMI exposure (Carmichael et al. 2014; Keil et al. 2014; Yang et al. 2014). Only Marfo et al. (2015) reported findings related to other neonics and their metabolites.

Of the associations reported, two were significant: between IMI and tetralogy of Fallot [Adjusted Odds Ratio (AOR) 2.4, 95% confidence interval (CI): 1.1, 5.4] (Carmichael et al. 2014) and between urinary DMAP and an increased prevalence of neurologic symptoms and 5 of 6 specific health measures (OR 14, 95% CI: 3.5, 57) (Marfo et al. 2015). Other findings included a weak association between IMI and ASD [AOR 1.3, 95% credible interval (CrI): 0.78, 2.2] (Keil et al. 2014), which became significant when the analysis was limited to self-reported frequent users (AOR 2.0, 95% CrI: 1.0, 3.9). The OR for ASD was higher for IMI exposures during the prenatal period versus during the first 3 years of life, although the finding was not significant (Keil et al. 2014). A suggestive association between IMI and anencephaly was also reported (AOR 2.9, 95% CI: 1.0, 8.2) (Yang et al. 2014).

## Discussion

To our knowledge, the present systematic review is the first to summarize the human health effects of exposure to neonics in the peer-reviewed literature. In the present review, eight studies were identified: four examining the health outcomes of acute neonic exposure and four examining the health effects of chronic (nonacute) neonic exposure.

### Acute Exposure Study Limitations

There were many differences among the acute neonic exposure studies that made further analysis and synthesis of their findings difficult.

***Study design and goals.*** Study designs and goals differed, with two retrospective studies (Forrester 2014; Phua et al. 2009) examining neonic poisoning cases and two prospective studies differing in both design and goals (Elfman et al. 2009; Mohamed et al. 2009). Mohamed et al. (2009) followed clinical outcomes and tracked the toxicokinetics of IMI following acute self-poisonings. The other prospective study, Elfman et al. (2009), had a double blind crossover design and focused on occupational exposure to neonics.

***Population differences.*** Sample sizes varied, from 19 planters in Elfman et al. (2009) to < 70 cases (Phua et al. 2009; Mohamed et al. 2009) to 1,142 cases (Forrester 2014). The *n* in Elfman et al. (2009) may have been too small to detect IMI health effects, biasing results to the null.

The distribution of age varied significantly among the studies, with children < 19 years comprising 37% of the cases in Forrester (2014) compared to no children < 14 years of age enrolled in Mohamed et al. (2009) and only two children included among the cases in Phua et al. (2009). The higher proportion of children (37%) and of non-intentional versus intentional ingestion cases in Forrester (2014) may account in part for the low rate of adverse health effects, as compared to Phua et al. (2009) and Mohamed et al. (2009). An adult with suicidal intent is likely to ingest a greater amount of neonic than a child.

The median age of ingestion cases was 54 in Phua et al. (2009); however, the average age differed significantly between the severely symptomatic group versus those who were asymptomatic or had mild to moderate symptoms, reported as 67 versus 49 respectively (*p* = 0.008). Again, age seemed to mediate the IMI findings. Underlying health conditions associated with age may have likewise mediated IMI case severity/fatality in the two poisoning studies reporting higher rates of adverse health effects (Phua et al. 2009; Mohamed et al. 2009).

The prevalence of self-poisoning versus accidental ingestion also differed significantly among the studies: less than 2% of the 1,142 cases examined by Forrester (2014) were considered intentional poisonings, in contrast to Phua et al. (2009), in which 81% of the cases were coded as suicide attempts, and Mohamed et al. (2009), in which 82% were confirmed (by the patient or a relative) as suicide attempts. Similarly, only 51% of the cases in Forrester (2014) were ingestions versus 91% (61 of 68) in Mohamed et al. (2009) and 81% (57 of 70) in Phua et al. (2009). The proportion of males to females also differed among the studies: 77% of the neonic cases in Phua et al. were male, 64% of the cases in Forrester (2014) were female. Mohamed et al. (2009) did not report a median age or gender. Data collection timeframes overlapped among the studies, but differed widely in number of years included. Forrester (2014) examined cases reported between 2000 and 2012, Phua et al. (2009) between 1987 and 2007, and Mohamed et al. (2009) from March 2002 to March 2007. It is therefore not surprising that findings focused more heavily on IMI since this was the main neonic in use during the majority of study years.

***Exposure/outcome assessment differences.*** Differences in exposure assessment methods may have contributed to the widely varying findings reported by the acute exposure studies. Phua et al. (2009) noted exposure was sometimes originally reported in number of mouthfuls, which the researchers quantified by considering one mouthful equal to 25 mL per for an adult or 9 mL per mouthful for a child. Mohamed et al. (2009) used biomonitoring (blood serum) to measure the amount of IMI ingested on presentation as well as to analyze absorption and elimination rates. The median amount of neonic ingested ranged from 15 mL (Mohamed et al. 2009) to 90 mL (range 50–200 mL) (Phua et al. 2009). Forrester (2014) did not provide any exposure (dose) data. Regarding outcome assessment, all four studies relied at least in part on interview data, with none reporting validation of questionnaires for internal consistency or factor loading or inter-rater agreement among interviewers.

Elfman et al. (2009) suffered several assessment limitations that may have contributed to its lack of findings for IMI-related health effects. The amount of IMI that planters were exposed to per seedling was quite low (1% pesticide formulation) compared to formulations reported in the other acute studies, which ranged from 9% to 17%. Exposure could occur via several pathways (dermal, inhalation, ingestion), but it was unclear why a 1-week time frame was considered long enough for an acute (but nonpoisonous) cumulative exposure to take place. The data were generated in part from biomonitoring (urine and nasal secretions). Given the lack of a validated biomarker, however, the urine results did not pertain to IMI. The nasal secretions were monitored for inflammatory response, which could be the result of other variables. Elfman et al. (2009) also noted the evaluation procedure itself may have affected results during the first week, with a drop off in awareness in weeks 2–3. The questionnaires were translated into Polish for 7 of 19 subjects, adding another source of information bias if the translation was not culturally competent. Although Elfman et al. (2009) controlled for serial correlation within each planter and exposure, the study did not report controlling for several potential external confounders, such as exposure to other pesticides, pollen, and differences in weather conditions experienced by planters.

### Chronic Exposure Study Limitations

The four chronic exposure studies (Carmichael et al. 2014; Keil et al. 2014; Marfo et al. 2015; Yang et al. 2014) shared a focus on associations between neonics and developmental outcomes. They shared similar limitations, most of which were related to case–control design.

***Sample size.*** Small sample size can limit precision and increase the possibility of Type II (false negative) errors. This was a possibility with all of the case–control studies. Of the 101 cases of tetralogy of Fallot, only 9 cases were exposed to IMI (Carmichael et al. 2014). In the NTD study, only 6 cases of anencephaly were exposed to IMI (Yang et al. 2014). The largest sample, 407 cases of ASD, was stratified for a Bayesian analysis in an effort to correct for exposure misclassification and recall bias. As a result, the number of individuals in some strata was “few” (Keil et al. 2014). Future studies should strive to increase the sample size, taking into account the desired statistical power, effect size, and the background prevalence of the outcome of interest (Perry 2008).

***Exposure assessment.*** All three developmental studies stated their findings could be the result of chance due to a large number of multiple comparisons (Carmichael et al. 2014; Yang et al. 2014) or exposure misclassification (Keil et al. 2014). Each relied heavily on pre-existing maternal interview data, introducing the possibility of recall or interviewer bias. None followed up with subjects individually or conducted biomonitoring.

The exposure assessment methods in Keil et al. (2014) differed from the other two developmental studies in several ways, including its focus on IMI alone as the main pesticide exposure; the exposure pathway (self-application of IMI in flea and tick products versus maternal residence proximity to agricultural pesticide use); and the statistical methods used to analyze data. Bayesian and frequentist analyses (versus logistic regression) were conducted to estimate the association between ASD and IMI in an effort to correct for both potential differential exposure misclassification and recall bias. The latter was of particular concern because the Childhood Autism Risks from Genetics and Environment (CHARGE) interview data were based on maternal recall of household pesticide use from, on average, 4 years in the past.

Exposure assignment differed among the studies as well. Keil et al. (2014) was based on monthly application of a flea and tick product containing IMI (Advantage and K9 Advantix, which contain ~ 9% IMI) from 3 months before conception, each trimester of pregnancy, and each year of the child’s life up to age 2. Carmichael et al. (2014) and Yang et al. (2014) assigned a time window for pesticide exposure corresponding to 1-month prior to or 2 months post conception. They estimated pesticide exposure based on data from the California Department of Pesticide Regulation, which described daily applications for the 461 pesticides studied (23,883,704 over the 10-year study period). Land-use survey field polygons provided by the California Department of Water Resources were spatially matched to pesticide use records. Temporal proximity was determined by comparing recorded dates of applications to the time window of exposure per each subject. Pounds of pesticides used within a 500-m radius of each subject’s geocoded address during the relevant window were calculated. Exposure was then assigned dichotomously (any or none). Of note, pesticide distribution within each polygon was assumed to be homogenous, and risks were not estimated for pesticides that had fewer than five exposed cases or controls, which could have weakened or missed associations.

***Confounding.*** The chronic exposure studies did not control for potential causes of birth defects or neurological and other symptoms, including the use of pesticides and other chemicals at home or work (Carmichael et al. 2014; Keil et al. 2014; Marfo et al. 2015; Yang et al. 2014). Carmichael et al. (2014) and Yang et al. (2014) reported they did not control for covariates that could have caused exposure misclassification, such as chemical half-lives, vapor pressure, wind patterns, and individual metabolic variability. Carmichael et al. (2014) and Yang et al. (2014) included several classes of pesticides but noted they did not correct results for multiple comparisons, increasing the potential for type 1 (false positive) error. Keil et al. (2014) did not control for air pollution, which is considered a possible risk factor for ASD. Ideally, future neonic-human health studies should strive to be more comprehensive in controlling for environmental and genetic factors as potential confounders or effect modifiers.

### Biomonitoring Data—All Studies

The eight studies varied widely in design, but all suffered from the lack of a validated biomarker for neonic exposure. A validated biomarker for IMI would enable more accurate exposure assessment (Elfman et al. 2009; Keil et al. 2014), greater understanding of metabolite production (Marfo et al. 2015; Phua et al. 2009), greater understanding of absorption and elimination variability (Marfo et al. 2015; Mohamed et al. 2009), and improved sensitivity testing to rule out false-positive results (Keil et al. 2014). The development of biomarkers for the most heavily used neonics and their metabolites would greatly assist future neonic-human health investigations.

### Research Implications

Limitations of this review include the possibility of missing data (studies published in languages other than English) and potential publication bias. Studies indicating null or weak but inconclusive associations between a neonicotinoid pesticide and a human health outcome may not have made it to publication, biasing the literature (Easterbrook et al. 1991; Franco et al. 2014; Nakagawa 2004).

To strengthen the internal validity of future studies, investigators should attempt to *a*) improve focus on neonics, both as a class and individually, rather than on mixtures of pesticides that include neonics; *b*) include drinking water and food sampling, air and household dust sampling, biomonitoring data (urine, serum), using validated biomarkers, if available, to provide a quantified, comprehensive, and environmentally relevant picture of neonic exposure; *c*) ensure adequate statistical power to detect associations; and *d*) control for potential confounders and effect modifiers, such as air pollution.

## Conclusions

To the authors’ knowledge, this is the first systematic review of the literature on human health effects of neonicotinoids. As reviewed here, four studies reported low rates of adverse health effects from acute neonic exposure. Even the most severe outcomes, including two fatalities, may have been mediated by other factors (age, underlying health conditions, undetected coexposures). The acute poisoning studies did, however, elucidate clinical findings important for the diagnosis and treatment of acute neonic exposures, including a better understanding of neonic toxicokinetics in humans. The other four studies reported associations between chronic neonic exposure and adverse developmental outcomes or a symptom cluster including neurological effects. The findings of animal studies support the biological plausibility for such associations (Abou-Donia et al. 2008; Gibbons et al. 2015; Gu et al. 2013; Kimura-Kuroda et al. 2012; Li et al. 2011; Mason et al. 2013; Tomizawa 2004).

Although the studies in this review represent an important contribution to the literature, particularly given the lack of any general population chronic exposure studies prior to 2014, there remains a paucity of data on neonic exposure and human health. Given the widespread use of neonics in agriculture and household products and its increasing detection in U.S. food and water, more studies on the human health effects of chronic (non-acute) neonic exposure are needed.

## Supplemental Material

(144 KB) PDF
