# Supplementary material for: Effects of Neonicotinoid Pesticide Exposure on Human Health: A Systematic Review
Source: Environ Health Perspect. 2016 Jul 6;125(2):155–62. doi: 10.1289/EHP515 (PMC5289916; doi:10.1289/EHP515)
Supplement: (144 KB) PDF [file EHP515.s001.acco.pdf]

Environ Health Perspect
DOI: 10.1289/EHP515

Note to readers with disabilities: *EHP* strives to ensure that all journal content is accessible to all readers. However, some figures and Supplemental Material published in *EHP* articles may not conform to 508 standards due to the complexity of the information being presented. If you need assistance accessing journal content, please contact ehp508@niehs.nih.gov. Our staff will work with you to assess and meet your accessibility needs within 3 working days.

Supplemental Material

Effects of Neonicotinoid Pesticide Exposure on Human Health: A Systematic Review

Andria M. Cimino, Abee L. Boyles, Kristina A. Thayer, and Melissa J. Perry

Table of Contents

1. Neonics and Human Health: All References
2. Abbreviations

1. Neonics and Human Health: All References (89) 12/14/15 (* indicates study retained for review)

(1) Abou-Donia AB, Goldstein LB, Bullman S et al. Imidacloprid induces neurobehavioral deficits and increases expression of glial fibrillary acidic protein in the motor cortex and hippocampus in offspring rats following in utero exposure. *Journal of toxicology and environmental health.Part A* 2008 -01-01;71(1-2):119; 119-130; 130.

(2) Alaux C. Interactions between microspores and a neonicotinoid weaken honeybees ( ). *Environ.Microbiol.* 2010 -03;12(3):774; 774-782; 782.

(3) Al-Taher F. Reduction of Pesticide Residues in Tomatoes and Other Produce. *J.Food Prot.* 2013 -03;76(3):510; 510-515; 515.

(4) Anderson JC, Dubetz C, Palace VP. Neonicotinoids in the Canadian aquatic environment: A literature review on current use products with a focus on fate, exposure, and biological effects. *Sci.Total Environ.* 2015;505:409-422.

(5) Aprea C. Evaluation of inhaled and cutaneous doses of imidacloprid during stapling ornamental plants in tunnels or greenhouses. *Journal of exposure science & environmental epidemiology* 2009 -09;19(6):555; 555-569; 569.

(6) Aprea MC. Environmental and biological monitoring in the estimation of absorbed doses of pesticides. *Toxicol.Lett.* 2012 APR 25;210(2):110-118.

(7) Bal R. Assessment of imidacloprid toxicity on reproductive organ system of adult male rats. *Journal of environmental science and health.Part B, Pesticides, food contaminants, and agricultural wastes* 2012 -05;47(5):434; 434-444; 444.

(8) Botton M. IPM on peaches in Brazil: Actual situation and future trends. *Acta horticulturae* 2002;592:655; 655-658; 658.

(9) Broznić D. Imidacloprid - olive orchard "guardian" | Imidakloprid - "čuvar" maslinika. *Medicina* 2009;45(2):119; 119-126; 126.

(10) Brunet J-, Maresca M, Fantini J, Belzunces LP. Intestinal absorption of the acetamiprid neonicotinoid by Caco-2 cells: Transepithelial transport, cellular uptake and efflux. *Journal of Environmental Science and Health - Part B Pesticides, Food Contaminants, and Agricultural Wastes* 2008;43(3):261-270.

(11) Calderón-Segura ME. Evaluation of Genotoxic and Cytotoxic Effects in Human Peripheral Blood Lymphocytes Exposed to Neonicotinoid Insecticides News. *Journal of toxicology* 2012;17(2):1; 1; 612647-11; 11.

(12) Cao L, Chen B, Zheng L, Wang D, Liu F, Huang Q. Assessment of potential dermal and inhalation exposure of workers to the insecticide imidacloprid using whole-body dosimetry in China. *J.Environ.Sci.(China)* 2015 Jan 1;27:139-146.

(13) Carmichael SL, Yang W, Roberts E, Kegley SE, Padula AM, English PB, et al. Residential agricultural pesticide exposures and risk of selected congenital heart defects among offspring in the San Joaquin Valley of California. *Environ.Res.* 2014 11;135(0):133-138.*

(14) Casida JE. Neonicotinoid metabolism: compounds, substituents, pathways, enzymes, organisms, and relevance. *J Agric Food Chem* 2011;59(7):2923-2931.

(15) Chen M. Quantitative analysis of neonicotinoid insecticide residues in foods: implication for dietary exposures. *J.Agric.Food Chem.* 2014 -07-02;62(26):6082.

(16) Choi H. Assessment of the Exposure of Workers to the Insecticide Imidacloprid during Application on Various Field Crops by a Hand-Held Power Sprayer. *J.Agric.Food Chem.* 2013 -11;61(45):10642; 10642-10648; 10648.

(17) Chwaluk P. [Acute inhalation imidacloprid poisoning--a case report]. | Ostre wziewne zatrucie imidachloprydem--opis przypadku. *Przeglad lekarski* 2010;67(8):619; 619-620; 620.

(18) Costa C. Cytogenetic and molecular biomonitoring of a Portuguese population exposed to pesticides. *Mutagenesis* 2006 -09;21(5):343; 343-350; 350.

(19) Costa C, Silvari V, Melchini A, Catania S, Heffron JJ, Trovato A, et al. Mutation Research/Genetic Toxicology and Environmental Mutagenesis. *Mutat.Res.-Genet.Toxicol.Environ.Mutag.* 2009 JAN 10;672(1):40-44.

(20) Craig M, Gupta R, Candery T, Britton D. Human exposure to imidacloprid from dogs treated with advantage. *Toxicol.Mech.Methods* 2005 JUL-AUG;15(4):287-291.

(21) Cressey D. Europe debates risk to bees. *Nature (London)* 2013 -04;496(7446):408; 408-408; 408.

(22) de Oliveira IM et al. Effects of the neonicotinoids thiametoxam and clothianidin on in vivo dopamine release in rat striatum. *Tox Ltrs* 2010;192:294.

(23) Deihimfard R, Soufizadeh S, Moinoddini SS, Kambouzia J, Zand E, Damghani AM, et al. Evaluating risk from insecticide use at the field and regional scales in Iran. *Crop Prot.* 2014 NOV;65:29-36.

(24) Dikshit AK, Pachauri DC, Jindal T. Maximum residue limit and risk assessment of beta-cyfluthrin and imidacloprid on tomato (Lycopersicon esculentum Mill). *Bull.Environ.Contam.Toxicol.* 2003 06;70(6):1143-1150.

(25) Ding F, Peng W. Biological assessment of neonicotinoids imidacloprid and its major metabolites for potentially human health using globular proteins as a model. *J.Photochem.Photobiol.B.* 2015 Mar 23;147:24-36.

(26) Elfman L, Hogstedt C, Engvall K, Lampa E, Lindh CH. Acute Health Effects on Planters of Conifer Seedlings Treated with Insecticides. *Ann.Occup.Hyg.* 2009 JUN;53(4):383-390.*

(27) Feng S. Assessing the genotoxicity of imidacloprid and RH-5849 in human peripheral blood lymphocytes in vitro with comet assay and cytogenetic tests. *Ecotoxicol.Environ.Saf.* 2005 -06;61(2):239; 239-246; 246.

(28) Ford K, Casida J. Unique and common metabolites of thiamethoxam, clothianidin, and dinotefuran in mice. *Chem.Res.Toxicol.* 2006 -11-20;19(11):1549; 1549-1556; 1556.

(29) Forrester MB. Neonicotinoid insecticide exposures reported to six poison centers in Texas. *Hum.Exp.Toxicol.* 2014 06;33(6):568-573.*

(30) Gawade L. A detailed study of developmental immunotoxicity of imidacloprid in Wistar rats. *Food and chemical toxicology* 2013 -01;51(1):61; 61-70; 70.

(31) Gibbons D, Morrissey C, Mineau P. A review of the direct and indirect effects of neonicotinoids and fipronil on vertebrate wildlife. *Environ.Sci.Pollut.Res.Int.* 2014 06/18.

(32) Gu Y, Li Y, Huang X, Zheng J, Yang J, Diao H, et al. Reproductive Effects of Two Neonicotinoid Insecticides on Mouse Sperm Function and Early Embryonic Development In Vitro. *PLoS One* 2013 _b 07_c 29;8(7):Article.

(33) Harris SA, Villeneuve PJ, Crawley CD, Mays JE, Yeary RA, Hurto KA, et al. National Study of Exposure to Pesticides among Professional Applicators: An Investigation Based on Urinary Biomarkers. *J.Agric.Food Chem.* 2010 SEP 22;58(18):10253-10261.

(34) Hladik ML, Kolpin DW, Kuivila KM. Widespread occurrence of neonicotinoid insecticides in streams in a high corn and soybean producing region, USA. *Environmental Pollution* 2014 10;193(0):189-196.

(35) Hou R. Comparison of the dissipation behaviour of three neonicotinoid insecticides in tea. *Food additives & contaminants.Part A, Chemistry, analysis, control, exposure & risk assessment* 2013 -10;30(10):1761; 1761-1769; 1769.

(36) Huseth AS, Groves RL. Environmental Fate of Soil Applied Neonicotinoid Insecticides in an Irrigated Potato Agroecosystem. *PLoS ONE* 2014 05;9(5):1-11.

(37) Ichikawa H. Neurotoxicology of pesticides. *Brain and Nerve* 2015;67(1):39-48.

(38) Itoiz ES. Deposition and residues of azoxystrobin and imidacloprid on greenhouse lettuce with implications for human consumption. *Chemosphere (Oxford)* 2012 -11;89(9):1034; 1034-1041; 1041.

(39) Kalajdzic P. Use of Mutagenesis, Genetic Mapping and Next Generation Transcriptomics to Investigate Insecticide Resistance Mechanisms. *PloS one* 2012 -06;7(6):e40296; e40296.

(40) Kamel F, Umbach DM, Bedlack RS, Richards M, Watson M, Alavanja MC, et al. Pesticide exposure and amyotrophic lateral sclerosis. *Neurotoxicology* 2012 Jun;33(3):457-462.

(41) Kapoor U, Srivastava MK, Srivastava AK, Patel DK, Garg V, Srivastava LP. Analysis of imidacloprid residues in fruits, vegetables, cereals, fruit juices, and baby foods, and daily intake estimation in and around Lucknow, India. *Environ.Toxicol.Chem.* 2013 Mar;32(3):723-727.

(42) Karabay NU. Cytogenetic and genotoxic effects of the insecticides, imidacloprid and methamidophos. *Genetics and molecular research* 2005;4(4):653; 653-662; 662.

(43) Kavvalakis MP. Development and application of LC–APCI–MS method for biomonitoring of animal and human exposure to imidacloprid. *Chemosphere (Oxford)* 2013 -11;93(10):2612; 2612-2620; 2620.

(44) Keil A, Daniels J, Hertz-Picciotto I. Autism spectrum disorder, flea and tick medication, and adjustments for exposure misclassification: the CHARGE (CHildhood Autism Risks from Genetics and Environment) case-control study. *Environ.Health* 2014;13(1):3.*

(45) Khan DA, Bhatti MM, Khan FA, Naqvi ST, Karam A. Adverse Effects of Pesticides Residues on Biochemical Markers in Pakistani Tobacco Farmers. *Int.J.Clin.Exp.Med.* 2008;1(3):274-282.

(46) Khan DA, Hashmi I, Mahjabeen W, Naqvi TA. Monitoring health implications of pesticide exposure in factory workers in Pakistan. *Environ.Monit.Assess.* 2010 SEP;168(1-4):231-240.

(47) Kim J. Imidacloprid, a neonicotinoid insecticide, induces insulin resistance. *J.Toxicol.Sci.* 2013;38(5):655; 655-660; 660.

(48) Kimura-Kuroda J, Komuta Y, Kuroda Y, Hayashi M, Kawano H. Nicotine-Like Effects of the Neonicotinoid Insecticides Acetamiprid and Imidacloprid on Cerebellar Neurons from Neonatal Rats. *PLoS One* 2012 _b 02_c 29;7(2):Article.

(49) Kocaman AY, Topaktas M. Genotoxic effects of a particular mixture of acetamiprid and a-cypermethrin on chromosome aberration, sister chromatid exchange, and micronucleus formation in human peripheral blood lymphocytes. *Environ.Toxicol.* 2010;25(2):157-168.

(50) Kozmutza C. To address accuracy and precision using methods from analytical chemistry and computational physics. *Environ.Monit.Assess.* 2009 -04;151(1-4):59; 59-75; 75.

(51) Krupke CH, Hunt GJ, Eitzer BD, Andino G, Given K. Multiple Routes of Pesticide Exposure for Honey Bees Living Near Agricultural Fields. *PLoS ONE* 2012 01;7(1):1-8.

(52) Kurwadkar ST, Dewinne D, Wheat R, McGahan DG, Mitchell FL. Time dependent sorption behavior of dinotefuran, imidacloprid and thiamethoxam. *Journal of Environmental Science & Health, Part B -- Pesticides, Food Contaminants, & Agricultural Wastes* 2013 03;48(4):237-242.

(53) Li P, Ann J, Akk G. Activation and Modulation of Human alpha 4 beta 2 Nicotinic Acetylcholine Receptors by the Neonicotinoids Clothianidin and Imidacloprid. *J.Neurosci.Res.* 2011 -08-01;89(8):1295; 1295-1301; 1301.

(54) Lopez-Antia A. Experimental exposure of red-legged partridges (Alectoris rufa) to seeds coated with imidacloprid, thiram and difenoconazole. *Ecotoxicology (London)* 2013 -01;22(1):125; 125-138; 138.

(55) Lozowicka B. Health risk for children and adults consuming apples with pesticide residue. *Sci.Total Environ.* 2015;502:184-198.

(56) Lu C, Schenck FJ, Pearson MA, Wong JW. Assessing Children's Dietary Pesticide Exposure: Direct Measurement of Pesticide Residues in 24-Hr Duplicate Food Samples. *Environ.Health Perspect.* 2010 11;118(11):1625-1630.

(57) Main AR, Headley JV, Peru KM, Michel NL, Cessna AJ, Morrissey CA. Widespread use and frequent detection of neonicotinoid insecticides in wetlands of Canada's Prairie Pothole Region. *PLoS One* 2014 03/26;9(3):e92821-e92821.

(58) Marfo JT, Fujioka K, Ikenaka Y, Nakayama SMM, Mizukawa H, Aoyama Y, et al. 2015. Relationship between Urinary *N*-Desmethyl-Acetamiprid and typical symptoms including neurological findings: a prevalence case-control study. PLoS ONE 10(11): e0142172. doi:10.1371/journal.pone.0142172.*

(59) Marin A, Martinez Vidal JL, Egea Gonzalez FJ, et al. Assessment of potential (inhalation and dermal) and actual exposure to acetamiprid by greenhouse applicators using liquid chromatography-tandem mass spectrometry. *Journal of chromatography.B, Analytical technologies in the biomedical and life sciences* 2004 -05-25;804(2):269; 269-275; 275.

(60) Matsuda K, Kanaoka S, Akamatsu M, Sattelle DB. Diverse actions and target-site selectivity of neonicotinoids: structural insights. *Mol.Pharmacol.* 2009 Jul;76(1):1-10.

(61) Mencke N. Acaricidal and repellent properties of permethrin, its role in reducing transmission of vector-borne pathogens. *Parassitologia* 2006;48(1-2):139; 139-140; 140.

(62) Mencke N. Therapy and prevention of parasitic insects in veterinary medicine using imidacloprid. *Current topics in medicinal chemistry* 2002;2(7):701; 701-715; 715.

(63) Mesnage R. Major Pesticides Are More Toxic to Human Cells Than Their Declared Active Principles. *Pathology* 2014;26(3):1; 1; 179691-8; 8.

(64) Mohamed F, Gawarammana I, Robertson TA, Roberts MS, Palangasinghe C, Zawahir S, et al. Acute Human Self-Poisoning with Imidacloprid Compound: A Neonicotinoid Insecticide. *PLoS ONE* 2009 04;4(4):1-5.*

(65) Mondal Sea. Impaired learning and memory after a week long exposure of acetamiprid in adult rats. *Adv Anim Vet Sci* 2014 October 30, 2014;2(10):543.

(66) Morrissey CA. Neonicotinoid contamination of global surface waters and associated risk to aquatic invertebrates: A review. *Environ.Int.* 2015 -01-01;74:291; 291-303; 303.

(67) Nomura H, Ueyama J, Kondo T, Saito I, Murata K, Iwata T, et al. Quantitation of neonicotinoid metabolites in human urine using GC-MS. *Journal of Chromatography B: Analytical Technologies in the Biomedical & Life Sciences* 2013 12/15;941:109-115.

(68) Phua DH, Lin CC, Wu M-, Deng J-, Yang C-. Neonicotinoid insecticides: An emerging cause of acute pesticide poisoning. *Clin.Toxicol.* 2009;47(4):336-341.*

(69) Pochi D. Potential Exposure of Bees, Apis mellifera L., to Particulate Matter and Pesticides Derived from Seed Dressing During Maize Sowing. *Bull.Environ.Contam.Toxicol.* 2012 -08;89(2):354; 354-361; 361.

(70) Ragas AMJ. Cumulative risk assessment of chemical exposures in urban environments. *Environ.Int.* 2011 -07;37(5):872; 872-881; 881.

(71) Sanchez-Bayo F. The trouble with neonicotinoids. *Science (New York, N.Y.)* 2014 -11;346(6211):806; 806-807; 807.

(72) Sattelle DB. Edit, cut and paste in the nicotinic acetylcholine receptor gene family ofDrosophila melanogaster. *Bioessays* 2005 -04;27(4):366; 366-376; 376.

(73) Sekeroglu V, Sekeroglu ZA, Demirhan E. Effects of commercial formulations of deltamethrin and/or thiacloprid on thyroid hormone levels in rat serum. *Toxicol.Ind.Health* 2014 02;30(1):40-46.

(74) Simoniello MF, Kleinsorge EC, Scagnetti JA, Grigolato RA, Poletta GL, Carballo MA. DNA damage in workers occupationally exposed to pesticide mixtures. *J.Appl.Toxicol.* 2008 NOV;28(8):957-965.

(75) SINGH J. Bacterial, Azotobacter, Actinomycetes, and Fungal Population in Soil after Diazinon, Imidacloprid, and Lindane Treatments in Groundnut (L.) Fields. *Journal of environmental science and health.Part B, Pesticides, food contaminants, and agricultural wastes* 2005 -09;40(5):785; 785-800; 800.

(76) Starner K, Goh KS. Detections of the neonicotinoid insecticide imidacloprid in surface waters of three agricultural regions of California, USA, 2010-2011. *Bull.Environ.Contam.Toxicol.* 2012 03;88(3):316-321.

(77) Sugeng AJ. Hazard-ranking of agricultural pesticides for chronic health effects in Yuma County, Arizona. *Sci.Total Environ.* 2013 -10;463-464:35; 35-41; 41.

(78) Taira K, Aoyama Y, Kawakami T, Kamata M, Aoi T. [Detection of chloropyridinyl neonicotinoid insecticide metabolite 6-chloronicotinic acid in the urine: six cases with subacute nicotinic symptoms]. *Chudoku kenkyu : Chudoku Kenkyukai jun kikanshi = The Japanese journal of toxicology* 2011;24(3):222-230.

(79) Taira K, Fujioka K, Aoyama Y. Qualitative profiling and quantification of neonicotinoid metabolites in human urine by liquid chromatography coupled with mass spectrometry. *PLoS One* 2013 11/12;8(11):e80332-e80332.

(80) Tanaka T. Effects of maternal clothianidin exposure on behavioral development in F1 generation mice. *Toxicol.Ind.Health* 2012 -09;28(8):697; 697-707; 707.

(81) Tennekes HA, Sánchez-Bayo F. 2011. Time-dependent toxicity of neonicotinoids and other toxicants: implications for a new approach to risk assessment. J Environ Anal Toxicol S:4. http://dx.doi.org/10.4172/2161-0525.S4-001.

(82) Tennekes HA, Sánchez-Bayo F. 2013. The molecular basis of simple relationships between exposure concentration and toxic effects with time. Toxicology 309:39-51. doi: 10.1016/j.tox.2013.04.007.

(83) Thompson TS. Pesticides in Fresh Potatoes Sold in Farmers' Markets in Alberta, Canada. *Bull.Environ.Contam.Toxicol.* 2011 -11;87(5):580; 580-585; 585.

(84) Tomizawa M. NEONICOTINOID INSECTICIDE TOXICOLOGY: Mechanisms of Selective Action. *Annu.Rev.Pharmacol.Toxicol.* 2005 -02;45(1):247; 247-268; 268.

(85) Tomizawa M. Imidacloprid, Thiacloprid, and Their Imine Derivatives Up-Regulate the α4β2 Nicotinic Acetylcholine Receptor in M10 Cells. *Toxicol.Appl.Pharmacol.* 2000 -11;169(1):114; 114-120; 120.

(86) Ueyama J. Biological monitoring method for urinary neonicotinoid insecticides using LC-MS/MS and its application to Japanese adults. *Journal of occupational health* 2014;56(6):461; 461-468; 468.

(87) van der Sluijs ,J.P., Amaral-Rogers V, Belzunces LP, Bijleveld van Lexmond ,M.F.I.J., Bonmatin J, Chagnon M, et al. Conclusions of the Worldwide Integrated Assessment on the risks of neonicotinoids and fipronil to biodiversity and ecosystem functioning. *Environ.Sci.Pollut.Res.Int.* 2014 10/10.

(88) Wang R. Highly sensitive and specific detection of neonicotinoid insecticide imidacloprid in environmental and food samples by a polyclonal antibody-based enzyme-linked immunosorbent assay. *J.Sci.Food Agric.* 2012 -04;92(6):1253; 1253-1260; 1260.

(89) Yang W, Carmichael SL, Roberts EM, Kegley SE, Padula AM, English PB, et al. Residential agricultural pesticide exposures and risk of neural tube defects and orofacial clefts among offspring in the San Joaquin Valley of California. *Am.J.Epidemiol.* 2014 Mar 15;179(6):740-748.*

2. Abbreviations

Neonic(s)	neonicotinoid(s)
ACE	acetamiprid
CLO	clothianidin
DMAP	*N*-desmethyl-acetamiprid, a metabolite of ACE
IMI	imidacloprid
nAChR	nicotinic acetylcholine receptor
THX	thiamethoxam
